# Kirschner Wire Prying and Leverage Technique: a new closed reduction method in treatment of pediatric “Irreducible Supracondylar Humerus Fractures”

**DOI:** 10.1186/s13018-024-04592-4

**Published:** 2024-02-02

**Authors:** Yuan Xiao, Arthur Clement, Chi Kang, Bo Ren, Xin Liu

**Affiliations:** 1 Department of Pediatric Orthopaedics, Sichuan Provincial Orthopaedic Hospital, No. 132 West First Section First Ring Road, Chengdu, 610041 Sichuan China; 2 Department of Otolaryngology Head and Neck Surgery, First Hospital of Shanxi Medical University, Taiyuan, 030000 Shanxi China

**Keywords:** Supracondylar Humerus Fractures, Pediatric, Open reduction, Closed reduction, Kirschner Wires

## Abstract

**Background:**

This study employs an innovative closed reduction approach to treat pediatric "Irreducible Supracondylar Humerus Fractures" with the goal of demonstrating its practical application compared to conventional methods.

**Methods:**

This study sampled 146 surgically treated cases of "Irreducible Supracondylar Humerus Fractures" in our department. After applying inclusion and exclusion criteria, 120 children were selected and divided into two groups based on treatment methods. Group 1 underwent Closed Reduction and Percutaneous Pinning (CRPP), while Group 2 received treatment using the Kirschner Wire Prying and Leverage Technique alongside CRPP. The relevant data to the study were collected and assessed during the follow-up period.

**Results:**

Results indicate that Group 2 demonstrated significantly shorter operative times and fewer instances of intraoperative fluoroscopy compared to Group 1. Furthermore, the percentage of cases requiring open reduction was notably higher in Group 1 than in Group 2. The analysis also identified age, BMI, time from injury to surgery, and the initial deviation of the distal fragment as independent risk factors associated with the failure of closed reduction.

**Summary:**

The integration of CRPP with the Kirschner Wire Prying and Leverage Technique emerges as a safe and effective strategy for managing "Irreducible Supracondylar Humerus Fractures." This innovative approach not only reduces operative time and intraoperative fluoroscopy needs but also diminishes the reliance on open reduction without compromising safety.

## Introduction

Supracondylar Humerus Fractures (SCFH) are common in children, accounting for 50% to 70% of elbow fractures, typically occurring between ages 3 and 10 [1, 2]. The primary surgical intervention for SCFH involves restoring anatomical alignment and maintaining stability [3]. Closed reduction and percutaneous pinning (CRPP) has traditionally been favored due to their ability to minimize complications [4, 5]. However, challenging cases may necessitate open reduction, accounting for 2% to 46% of SCFH cases, particularly in situations of fracture instability or severe displacement during motion [3, 6, 7]. The latter, referred to as "Irreducible Supracondylar Humerus Fractures" (ISHF), poses unique challenges, such as soft tissue impeding CRPP practicability [10, 11]. Recognizing this complex scenario, our study explores innovative treatment methods for ISHF.

In January 2020, our department introduced the Kirschner Wire Prying Technique, complemented by the Kirschner Wire Leverage Technique [12] and CRPP, as the safest and most effective method for treating ISHF. This technique addresses the significant challenge posed by Irreducible Supracondylar Humerus Fractures in pediatric orthopedics. Through extensive clinical practice, the Kirschner Wire Prying Technique, integrated with complementary methods, has shown promising results in achieving stable anatomical alignment [13–15]. Ongoing research and clinical validation aim to further refine these techniques for the benefit of pediatric patients facing this intricate orthopedic challenge [12, 15]. Contributing to medical practice and literature, our study applied the Kirschner Wire Leverage Technique in 120 children at our hospital, providing valuable insights into the innovative management of ISHF.

## Study background and context

Kirschner Wires, commonly known as K-Wires, are slender wires extensively utilized in orthopedic surgery to stabilize fractured bones [12]. These wires are delicately inserted through the skin and surrounding tissues, playing a crucial role in maintaining bone fragments in their proper positions during the healing process. Additionally, the Prying and Leverage Technique, a method employed in medical procedures to manipulate or position anatomical structures and medical devices, has emerged as a valuable approach [12]. This technique involves the strategic application of force or pressure in a controlled manner to achieve precise and predetermined outcomes [12, 15]. Leveraging these techniques allows healthcare professionals to navigate complex procedures effectively and optimize patient outcomes through careful manipulation and positioning.

It is noteworthy that the joint capsule and ligaments exhibit greater strength compared to bone, making them susceptible to fractures caused by external forces [14]. Metaizeau introduced percutaneous Elastic Stable Intramedullary Nailing (ESIN) fixation as a treatment method for such fractures [15]. However, achieving satisfactory reduction with the rotation technique of ESIN alone can be challenging for Judet type III [12]. The combined technique has shown promise in improving the quality of reduction [12, 15]. However, it is essential to note that this combined approach may increase surgical trauma, radiation exposure, operation time, and the risk of complications such as reduction failure, iatrogenic epiphyseal injury, premature epiphyseal closure, and radial nerve injury.

## Methods

### Data collection procedure

The study commenced by adhering to ethical protocols, obtaining approval from the hospital's Ethics Committee in the first week of January 2018. Between March 2018 and September 2021, a systematic collection of 146 cases of ISHF was conducted at the Emergency Department of our hospital, involving patients aged 4–14 years. Various parameters, including age, gender, BMI, initial deviation direction of the distal fragment, duration between injury and surgical intervention, surgical timing, intraoperative fluoroscopy instances, Bauman angle, alignment between the humeral front line and humeral head, and elbow function, were compiled for data analysis.

Resident physicians in our department acquired patients' fundamental information, while two senior pediatric orthopedic surgeons were responsible for identification, radiographic assessments of reduction quality, and ISHF procedures. The Gartland classification was determined based on preoperative and intraoperative X-rays, and the initial deviation of the distal fragment was assessed using original X-rays.

### Patient’s data summary and statistical analysis

The study employs inclusion and exclusion criteria. That is, after applying inclusion and exclusion technique, this study included 120 children, who were then categorized into two groups based on their respective treatment methods. Group 1 comprised 68 cases (55.7%) admitted to our hospital before January 2020 and treated with CRPP, while Group 2 consisted of the remaining 52 cases (44.3%) admitted after April 2020, receiving treatment with the Kirschner Wire Prying and Leverage Technique combined with CRPP. The exclusion criteria encompassed: (1) cases involving open injury, multiple injuries, neurovascular injury, or compartment syndrome. (2) Incomplete follow-up data. The numerical variables were presented as means and standard deviations, and frequencies and percentages were used for categorical data. Subgroup analyses utilized Chi-squared tests and Student's t tests, while logistic regression was employed to identify independent risk factors associated with CRPP failure. The statistical significance was considered at a *P* value < 0.05, and IBM SPSS Statistics ver. 23.0 (IBM Co., Armonk, NY, USA) was used for all data analyses.

### Surgical procedure using Kirschner Wire Prying and Leverage Technique

**Fig. 1:**
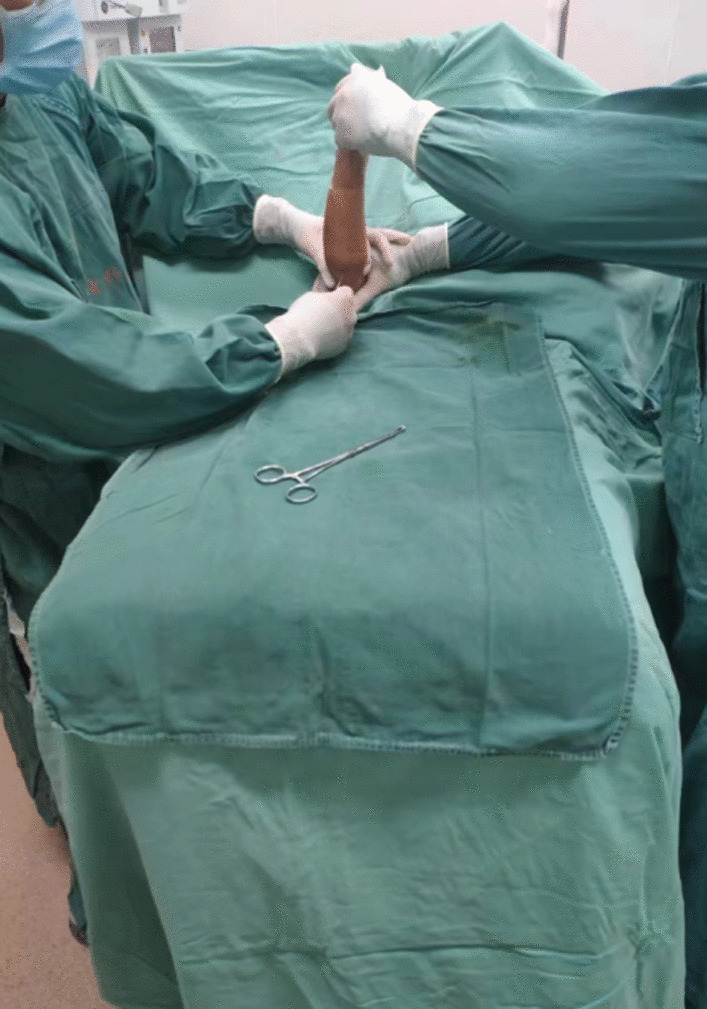
The position of surgeons and patient when doing the prying reduction. The assistant positioned the shoulder joint, and the surgeon, standing on the opposite side, pierced the skin with a 2.5-mm Kirschner wire, blunting the tail against the bone cortex above the olecranon fossa

**Fig. 2:**
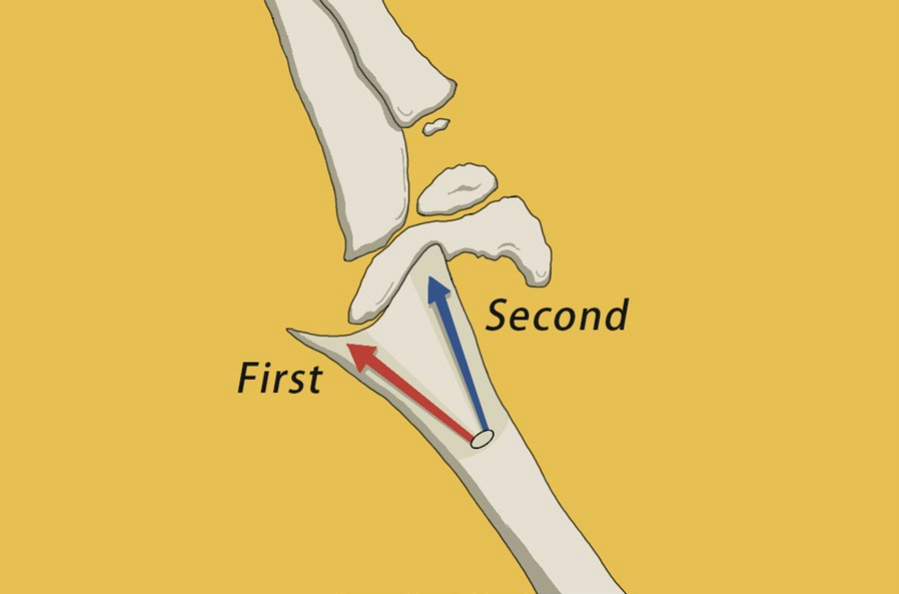
Diagrammatic sketch of Kirschner Wire Prying Technique (taking the distal fragment radial deviated SCHF as an example). The puncturing spot is about 0.5–1 cm above the olecranon fossa

**Fig. 3:**
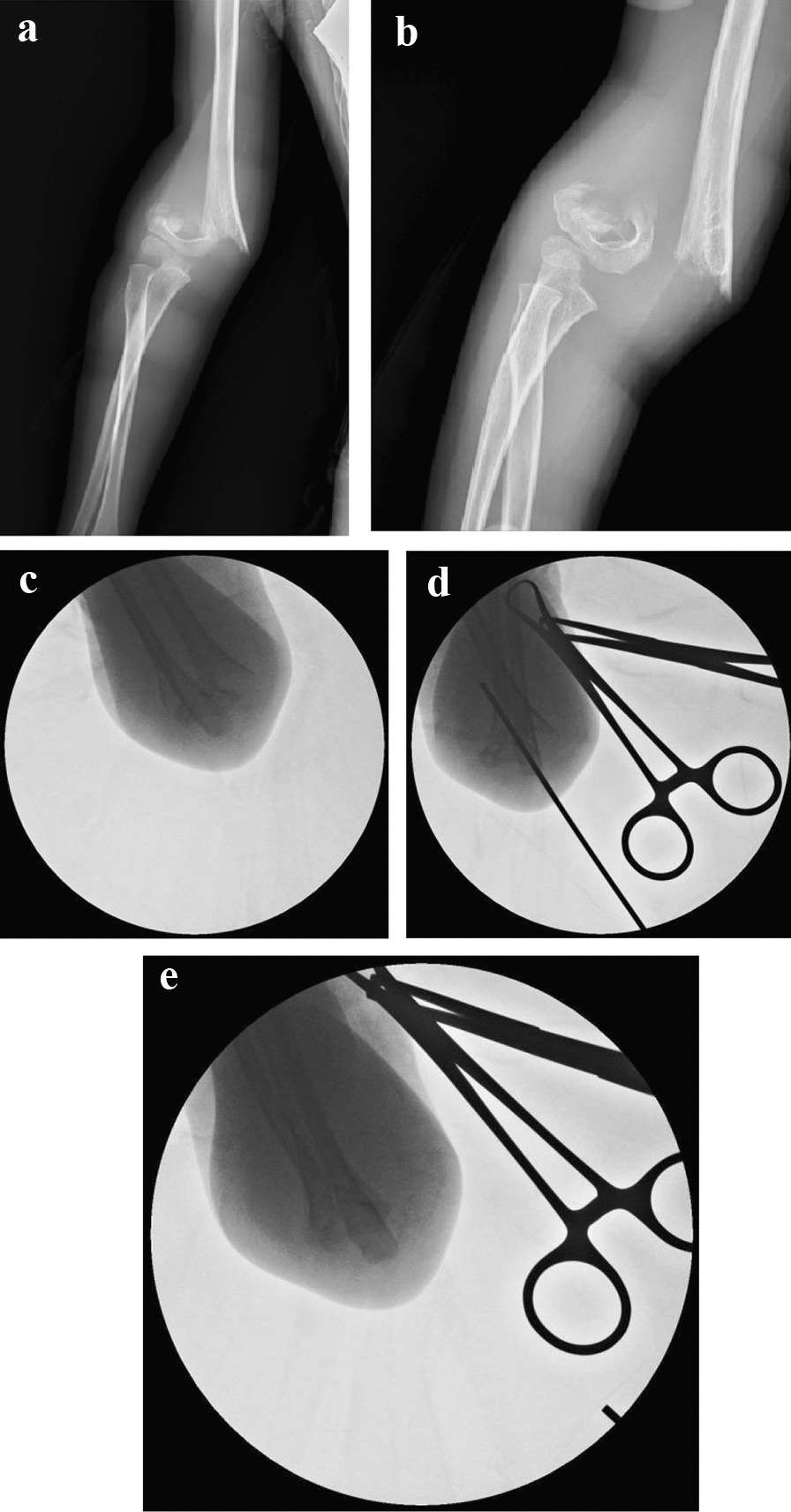
**a**–**e** The application of Kirschner Wire Prying Technique in practice (a type-IV SCFH in a 4.9-year-old boy). **a**, **b** Original X-ray of a posterolateral deviated SCFH. **c** After anesthesia, the surgeon could not make the position of fracture better by the gentle reduction. **d**, **e** The reduction gets better after using the Kirschner Wire Prying Technique on the AP view

Between January 2020 and April 2020, the Kirschner Wire Prying and Leverage Technique [12] was developed and implemented in conjunction with CRPP as part of this study. The criteria defining SCFH as "ISHF" included two key aspects: Patients under 14 years old diagnosed with Gartland type III and type IV SCFH. Following anesthesia, the surgeon's inability to detect bone friction feeling, hear bony crepitus, and/or generate significant displacement between the fracture ends during the single manual reduction procedure. The surgical procedure involved anesthesia and brachial plexus block anesthesia, positioning the child in a supine manner with the affected limb on the operating table to ensure the elbow was centered on the image intensifier. Sterile gloves were worn on the injured hand, and a sterile film was applied for sealing. The surgeon initiated a gentle reduction to confirm the absence of bone friction feeling, bony crepitus, and mutual movement between the fracture ends, thereby confirming the fracture as "ISHF." Subsequently, the assistant positioned the shoulder joint, and the surgeon, standing on the opposite side, pierced the skin with a 2.5-mm Kirschner wire [12], blunting the tail against the bone cortex above the olecranon fossa (Fig. 1). For distally deviated "ISHF," Kirschner Wire Prying occurred from proximal to distal along the ulnar ridge, followed by lateral ridge prying (Fig. 2) [14, 15]. In cases of ulnar deviation, radial prying preceded ulnar prying. Once the embedded soft tissue was relieved, the surgeon could perceive bone friction, bony crepitus, and movement between the fracture ends, signifying the transformation of a challenging-to-reduce fracture into a relatively manageable one (depicted in Fig. 3). Following the surgeon's assessment of the fracture's stability, if it presented as a simple extension type-III SCFH with an intact posterior periosteum hinge, the conventional closed reduction technique was employed.

**Fig. 4:**
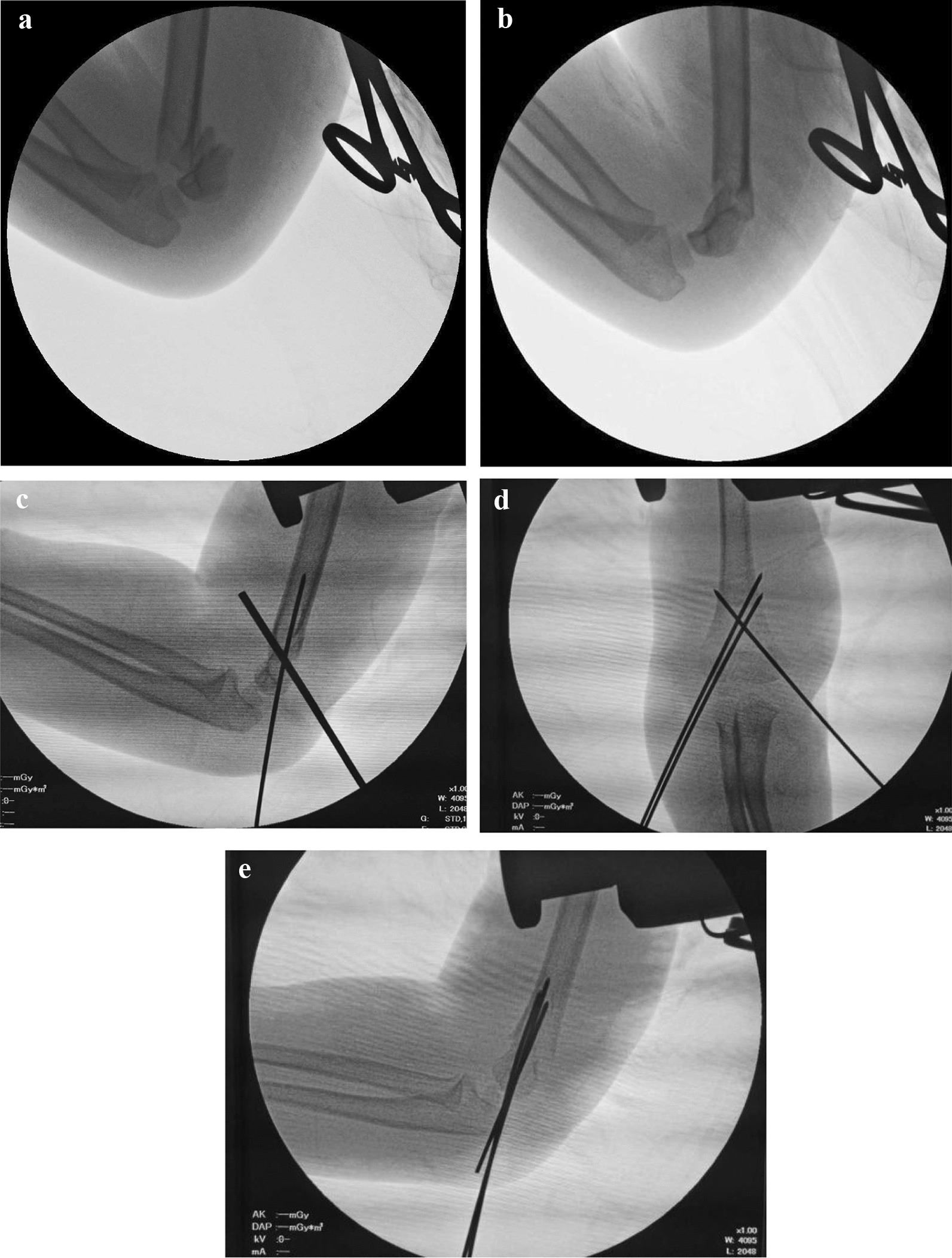
**a**–**e** The application of Kirschner wire leverage technique in practice. **a**, **b** We observed the fracture was unstable on the lateral view. **c** The deformity has been corrected by the Kirschner wire leverage technique, and a lateral pin has been fixed, making the fracture preliminary stable. **d**, **e** An acceptable reduction obtained by cross-Kirschner wire fixation

In cases of flexion or type-IV SCFH, the Kirschner wire leverage technique assisted in the reduction process. The tail of the 2.5-mm Kirschner wire was inserted into the fracture space, ensuring its extension beyond the anterior cortex of the humerus [12]. With gentle longitudinal traction from the assistant, the operator, holding the elbow, utilized the thumb and other fingers to execute push-and-pull maneuvers, correcting coronal plane deformities. The sagittal plane deformity was addressed through the lever effect of the Kirschner wire, maintaining the fracture reduction in a flexed position to facilitate lateral pin positioning (Fig. 4). Upon achieving an anatomical or acceptable bone position, the initial 1.5-mm lateral Kirschner wire, positioned near the central axis of the humerus, was placed. Due to potential instability, some reduction loss might occur after this placement [12]. Therefore, during the placement of the second lateral 1.5-mm Kirschner wire, the surgeon, assisted by another hand and the assistant, corrected any residual displacement of the fracture. Subsequently, the 2.5-mm Kirschner wire was removed, the elbow was extended, and the third 1.5-mm Kirschner wire was placed on the medial side, forming a cross-fixation with the lateral Kirschner wires. Following confirmation of an acceptable reduction through AP and lateral views of the image intensifier, the pins were cut short and bent over sterile felt padding [12].

In cases where closed reduction did not achieve satisfactory results, open reduction and cross-Kirschner wire fixations were performed [15]. The incision location was determined by the distal fracture's displacement direction: a medial approach for radial displacement and a lateral approach for ulnar displacement, with the aim of preserving the periosteum hinge and soft tissue integrity. The injured limb was immobilized with a polymer splint fixed at an elbow flexion of 60°. Regular follow-ups, occurring every 2 to 3 weeks for a minimum of 12 weeks, involved the removal of the polymer splint at the first visit, encouraging active elbow exercises. At the second follow-up, if X-rays indicated satisfactory fracture healing, the Kirschner wire was removed [12, 15]. Following the last follow-up of all cases, a comprehensive comparison between the two groups was conducted, considering operation time, intraoperative fluoroscopy times, percentage of open reduction, and quality of reduction, elbow function, and postoperative complications, among other factors.

## Results analysis

A total of 120 cases were monitored after the exclusion over an average follow-up period of 33.1 weeks (range 12–78 weeks). No significant difference was observed between Group 1 (mean: 35.6 weeks; range 12–78 weeks; SD ± 17.5 weeks) and Group 2 (mean: 29.7 weeks; range 12–71 weeks; SD ± 15.2 weeks) (p = 0.234). Group 1 comprised 43 males (63.2%) and 25 females (36.8%) with an average age of 7.83 years (range 4–14 years; SD ± 2.54 years), while Group 2 included 40 males (76.9%) and 12 females (23.1%) with an average age of 7.32 years (range 4–14 years; SD ± 2.00 years) (P = 0.064). The time to surgery, Gartland classification, and direction of the initial deviation of the distal fragment were essentially the same in both groups (Table 1). However, surgical time was significantly shorter in Group 2 (mean: 47.51 min; range 25–137 min; SD ± 24.16 min) compared to Group 1 (mean: 77.98 min; range 25–145 min; SD ± 36.96 min) (*P* < 0.01). The frequency of fluoroscopy in Group 2 (mean: 36.44 times; range 16–110 times; SD ± 20.2 times) was also significantly lower than in Group 1 (mean: 61.16 times; range 16–123 times; SD ± 29.8 times) (*P* < 0.01).

**Table 1:** Clinic characteristics of all patients (*n* = 120)

Characteristic	Group 1 (*n* = 68)	Group 2 (*n* = 52)	*P* value
Age (yr)	7.83 ± 2.54	7.32 ± 2.00	*P* = 0.064
Sex			
Male	43 (63.2%)	40 (76.9%)	*P* = 0.98
Female	25 (36.8.%)	12 (23.1%)	
BMI	17.22 ± 2.61	17.56 ± 2.73	*P* = 0.486
Time to surgery (d)	3.50 ± 1.67	2.75 ± 1.39	*P* = 0.073
Gartland classification			
Type3	19	12	*P* = 0.546
Type4	49	40	
Initial deviation			*P* = 0.465
Posterolateral (extension)	38	35	
Posteromedial (extension)	14	10	
Anterolateral (flexion)	15	6	
Anteromedial (flexion)	1	1	

In terms of the percentage of open reduction, Group 2 (5.8%) exhibited a much lower rate than Group 1 (35.3%) (*p* < 0.01) (Table 2). The Baumann angle on the AP radiographic view at the last follow-up was similar between the two groups, with Group 1 (mean BA: 72.4°; SD ± 5.0°) and Group 2 (mean BA: 70.5°; SD ± 4.8°) (P = 0.742). Elbow motion range recovery, compared with the contralateral side by percentile, was better in Group 2 (mean: 97.7%; SD ± 2.8%) than in Group 1 (mean: 95.3%; SD ± 5.8%) (*p* < 0.01) (Table 2). Complications in Group 1 included five cases of superficial pin site infection (7.3%), whereas Group 2 had only one case of pin infection (1.9%) (P = 0.174). No evidence of malunion, loss of reduction, iatrogenic nerve injury, or compartment syndrome was observed during surgical procedures. In the overall analysis, including both open and closed reduction groups, univariate analysis revealed a significant correlation between open reduction and age, BMI, time from injury to surgery, and the initial deviation of the distal fragment of the fracture, but not gender and Gartland classification (Table 3). In multivariate analysis, BMI, time to surgery, and initial deviation of the distal fragment were identified as independent risk factors for converting from closed to open reduction (Table 4).

**Table 2:** Comparison of intraoperative data and clinical outcomes between Group 1 and Group 2

Group	Number	Surgical time (minutes)	Times of fluoroscopy (times)	Percentage of open reduction (%)	Range of motion (%)	Baumann angle (°)
Group 1	68	77.98 ± 36.96	61.16 ± 29.8	35.3%	95.3 ± 5.8	72.4 ± 5.0
Group 2	52	47.51 ± 24.16	36.44 ± 20.2	5.8%	97.7 ± 2.8	70.5 ± 4.8
*P* value	*P* > 0.05	*P* < 0.01	*P* < 0.01	*P* < 0.01	*p* < 0.01	*P* > 0.05

**Table 3:** Summary of pertinent variables associated with or without open reduction (*n* = 120)

Characteristic	CRPP (*n* = 93)	ORIF (*n* = 27)	*P* value
Age (yr)			*P* < 0.001
4–6y	41 (97.6%)	1 (2.4%)	
7–9y	38 (72%)	15 (28%)	
10–14y	14 (56%)	11 (44%)	
Sex			*P* = 0.618
Male	60 (79%)	16 (21%)	
Female	33 (75%)	11 (25%)	
BMI	16.84		*P* = 0.002
< 17.37	55 (89%)	7 (11%)	
> 17.37	38 (66%)	20 (34%)	
Time to surgery (hs)			*P* = 0.007
< 24hs	17 (94.5%)	1 (5.5%)	
24–72hs	47 (84%)	9 (16%)	
> 72hs	29 (63%)	17 (37%)	
Gartland classification			*P* = 0.137
Type3	27 (87%)	4 (13%)	
Type4	66 (74%)	23 (26%)	
Initial deviation			*P* < 0.001
Posterolateral (extension)	60 (82%)	13 (18%)	
Posteromedial (extension)	23 (96%)	1 (4%)	
Anterolateral (flexion)	8 (38%)	13 (62%)	
Anteromedial (flexion)	2 (100%)	0 (0)

**Table 4:** Results of logistic regression analysis

Variable	Coefficient	SE	Wald	*P* value	RR (95%CI)
Age	.406	.120	11.532	.001	1.501 (1.187–1.898)
Time to surgery	.509	.171	8.820	.003	1.664 (1.189–2.329)
Initial deviation	− .749	.309	5.864	.015	0.473 (0.258–0.867)
BMI	1.331	.572	5.418	.020	3.786 (1.234–11.617)

## Discussion

SCFH is a prevalent pediatric elbow injury, and CRPP is generally considered the standard approach for displaced fractures, except in cases of specific complications such as severe neurologic disruption, vascular injury, open fractures, or compartment syndrome [16, 17]. Achieving anatomic reduction and stable fixation through CRPP is associated with favorable clinical and cosmetic outcomes [18, 19]. However, challenges may arise in some SCFH cases, leading to unsuccessful CRPP and necessitating open reduction. This, in turn, increases the risk of complications in children, including loss of motion, myositis ossificans, infection, and hypertrophic scars [20–24].

Apart from situations involving compartment syndrome, open fractures, and severe neurovascular injuries, two primary reasons warrant open reduction. First, the fracture may be inherently unstable, and second, soft tissue might enter the fracture gap or become trapped at the fracture end, complicating the reduction process. Leitch et al. [8] classified multidirectionally unstable SCFH as type-IV SCFH, indicating fractures unstable in both extension and flexion due to severe trauma causing circumferential disruption of the periosteum-a hinge maintaining reduction in type II and III fractures. Since the identification of this fracture type, scholars have conducted related studies on multiple unstable SCFH. Silva et al. [25] employed the towel roll and Kirschner wire joystick technique along with CRPP for 12 cases of type-IV supracondylar fractures, with open reduction required in 17% of cases. Ei et al. [26] introduced the "Kirschner wire leverage technique" combined with CRPP for 27 cases of type-IV SCFH, reporting a 100% rate of satisfactory outcomes, with 96.3% classified as good or excellent. Wei et al. [9] presented the transolecranon pin joystick technique for treating type-IV SCFH, demonstrating significantly reduced operation time and intraoperative fluoroscopy, improved fracture reduction quality on the anteroposterior radiographic view, and no postoperative complications compared to traditional CRPP. Studies by Novais et al. [3] and Reitman et al. [6] identified factors leading to fracture irreducibility, including instability, brachialis interposition, periosteal interposition, or triceps interposition. Archibeck et al. [11] reported that 90% of irreducible cases were associated with brachialis interposition.

The existing literature offers several effective and secure solutions for treating unstable SCFH using CRPP within the academic and health practicing community. However, there is a noticeable gap in studies focusing on the removal of soft tissue obstruction at the fracture's broken end to restore the pathway for closed reduction. Peters et al. [10] introduced a "milking maneuver technique" for type-III supracondylar fractures, demonstrating clinical and radiographic success. Nevertheless, uncertainty surrounds the technique's efficacy for patients with significant swelling lasting longer than 48 h. Suh et al. [27] treated 78 cases of type-III SCFH using minimal incisions and manipulation, reporting excellent outcomes in 76 cases and poor results in 2 cases after a follow-up of at least 3 years. However, these studies lack cases of type-IV SCFH.

In addressing ISHF, our approach involves assessing bone friction feeling and bony crepitus through a single manual reduction after anesthesia. Conventional CRPP is considered feasible if both aspects are achieved. However, bone friction feeling without significant mutual movement may indicate the fracture tip puncturing into the muscle, hindering reduction. If mutual movement occurs without bone friction feeling, periosteum, muscle, or other soft tissues may obstruct reduction, presenting a challenge. Blind manual reduction risks exacerbate soft tissue and neurovascular injuries. Consequently, we applied a Kirschner Wire Prying Technique to eliminate soft tissue obstruction, facilitating closed reduction and transforming an irreducible fracture into a reducible one. De Boeck et al. [28] highlighted challenges in treating type III flexion fractures with CRPP, especially when the elbow is extended, and introduced a Kirschner wire leverage technique to maintain unstable fractures in a maximally flexed position after reduction, stabilizing them through pinning. Lee et al. [29] introduced this leverage technique decades ago, achieving promising outcomes for type-III SCFH using designed and manufactured Steinmann pins. Applying this technique in the study, comparison to Group 1, Group 2 demonstrated a significant reduction in operative time and intraoperative fluoroscopy exposure through the utilization of the Kirschner Wire Prying and Leverage Technique. Simultaneously, Group 2 exhibited a markedly superior elbow functional range compared to Group 1. This improvement may be attributed to the considerably lower percentage of open reduction in Group 2 (3/52, 5.8%) compared to Group 1 (24/68, 35.3%). Open reduction has the potential to cause additional soft tissue injury and adhesion, contributing to the observed differences in functional outcomes. However, due to the limited follow-up duration in this study, confirmation of potential improvements in functional activity for children in Group 1 during later growth and development remains inconclusive. It is noteworthy that neither cubitus varus nor cubitus valgus was observed in any cases within this study, emphasizing the importance of the surgeon's meticulous control over the quality of reduction.

Subsequently, comparing cases of open reduction with those of closed reduction, a clear trend emerged, indicating an increased likelihood of open reduction with advancing age. In our investigation, the proportion of open reduction for children aged 4 to 6 stood at 2.4%, escalating to 28% for those aged 7–9 and reaching a substantial 44% for children aged 10–14. These findings align with those of Fletcher et al. [30], who reported a higher incidence of open reduction in patients older than 8, emphasizing the potential association with higher-energy mechanisms compared to younger cohorts. Farnsworth et al. [31] further highlighted that children older than 6 were more prone to injuries from elevated falls, contrasting with infants and toddlers who tended to experience falls from lower heights. Contrary to our study, Beck et al. [32] reported that age was not an independent risk factor for closed reduction failure. While our study did not unveil a statistically significant gender difference in the likelihood of open reduction, Novais et al. [3] observed a significant correlation between age, gender, and the risk of open reduction. Among females, a yearly age increase correlated with a 48% rise in the risk of open reduction, a trend not mirrored in males.

Moreover, this study identified a robust relationship between the time to surgery and the risk of open reduction. Instances of open reduction were 5.5% for patients undergoing surgery within 24 h, 16% for those within 24 to 72 h, and 36% for surgeries conducted beyond 72 h. We posit that progressive swelling in the injured limb postinjury continuously heightens the challenge of fracture reduction, leading to the failure of closed reduction and necessitating open reduction. A similar effect on the failure of closed reduction is believed to be associated with BMI. Although Garg S. et al. [33] reported a higher need for open reduction in patients taken to surgery within 6 h, our study did not establish a significant association with Gartland classification but did correlate with the initial deviation of the distal fragment. Specifically, 62% of anterolateral deviated fractures and 18% of posterolateral deviated fractures underwent open reduction. In contrast, posteromedial and anteromedial deviated fractures exhibited only 4% and 0% open reduction, respectively. This corresponds with findings by Novais et al. [3], who emphasized a strong link between flexion and posterolateral extension patterns and the likelihood of open reduction. The study suggested that the medial and posterior periosteal hinges are more robust than their anterior and lateral counterparts, rendering flexion and lateral deviated fractures more challenging and severe, consequently increasing the probability of open reduction. It is important to note that due to differing treatment methods in our study, these analyses may be influenced, prompting the need for prospective studies with meticulous data collection for a more accurate assessment of factors influencing successful closed reduction.

## Conclusion and recommendations and limitations

In conclusion, this study applied innovative technique, Kirschner Wire Prying and Leverage Technique in investigating this experiment. The study suggests that this technique presents itself as a promising and innovative solution for addressing ISHF. This technique was developed to overcome challenges encountered in fractures resistant to conventional closed reduction. The technique has demonstrated favorable outcomes in clinical practice. The significance of this approach is particularly evident in cases lacking bone friction feeling, audible bony crepitus, or significant displacement between fracture ends. Through a meticulous process involving initial manual reduction assessment under anesthesia, the technique allows for a personalized intervention tailored to the specific characteristics of each fracture. The study conducted comparative analysis between Group 1, utilizing traditional methods, and Group 2, benefitting from the Kirschner Wire Prying and Leverage Technique, revealed noteworthy advantages. Group 2 revealed reduced operative time, fewer instances of intraoperative fluoroscopy, and improved range of elbow function. These findings underscore the potential of the technique in minimizing the necessity for open reduction, thereby mitigating associated risks such as soft tissue injury.

## Recommendations

Consequently, recognizing the positive outcomes and potential benefits of the Kirschner Wire Prying and Leverage Technique, there is a need for healthcare institutions, researchers, and governing bodies to consider its inclusion in standardized protocols for managing ISHF. Policymakers should collaborate with medical professionals to develop practical guidelines that promote the integration of this innovative technique into orthopedic practices. Additionally, training programs and continuing education initiatives for orthopedic surgeons should incorporate instruction on the application of the Kirschner Wire Prying and Leverage Technique, ensuring widespread competence and proficiency in its utilization across healthcare settings. This proactive approach to policy development and dissemination will contribute to improved patient outcomes and enhanced efficiency in the management of ISHF.

## Limitations

Finally, this study has few limitations that warrant considerations. Firstly, it is important to acknowledge the retrospective nature of this study, with the two patient groups undergoing treatment at different time periods. Despite the inherent constraints associated with a retrospective design and the absence of a direct open reduction control group, the positive outcomes observed in both patient cohorts highlight the clinical significance and effectiveness of the Kirschner Wire Prying and Leverage Technique for ISHF. Secondly, the inclusion criteria for "ISHF" introduce an element of subjectivity, particularly in the description of situations where "the surgeon could not sense the bone friction feeling and hear the bony crepitus" or "the surgeon could not generate significant displacement between the fracture ends." While these criteria often align with the challenges encountered during surgery, this subjective nature introduces a potential limitation. Thirdly, the absence of a control group undergoing direct open reduction hinders the ability to draw conclusive comparisons with Group 1 and Group 2. This is especially pertinent for fractures with a high likelihood of open reduction. Establishing the superiority of our technique over direct open reduction necessitates a comparative analysis that was not implemented in this study. To therefore address these limitations and enhance the robustness of our findings, further prospective studies may incorporate a well-matched control group that provides more robust basis for evaluating the efficacy of this technique in comparison with alternative approaches, addressing these limitations to enhancing the overall reliability of the findings and larger sample sizes are warranted. Such studies would provide a more comprehensive validation of the observed positive outcomes and ascertain the broader applicability of the Kirschner Wire Prying and Leverage Technique in various clinical settings.

## Data Availability

The analyzed dataset in this study is available from the first author on reasonable request.
